# Correction: DAPE cloning with modified primers for producing designated lengths of 3’ single-stranded ends in PCR products

**DOI:** 10.1371/journal.pone.0344605

**Published:** 2026-03-09

**Authors:** Seoee Lee, Hyunyoung Kim, Aqsa, Kwangjin Jeung, Minho Won, Hyunju Ro

An additional affiliation is missing for the fourth author. Kwangjin Jeung is also affiliated with the Department of Applied Biological Engineering, Korea University of Scienceand Technology (UST), Daejeon, Korea (ROK).
