# Supplementary figures and images for: DAPE cloning with modified primers for producing designated lengths of 3’ single-stranded ends in PCR products
Source: PLoS One. 2025 Feb 13;20(2):e0318015. doi: 10.1371/journal.pone.0318015 (PMC11825038; doi:10.1371/journal.pone.0318015)

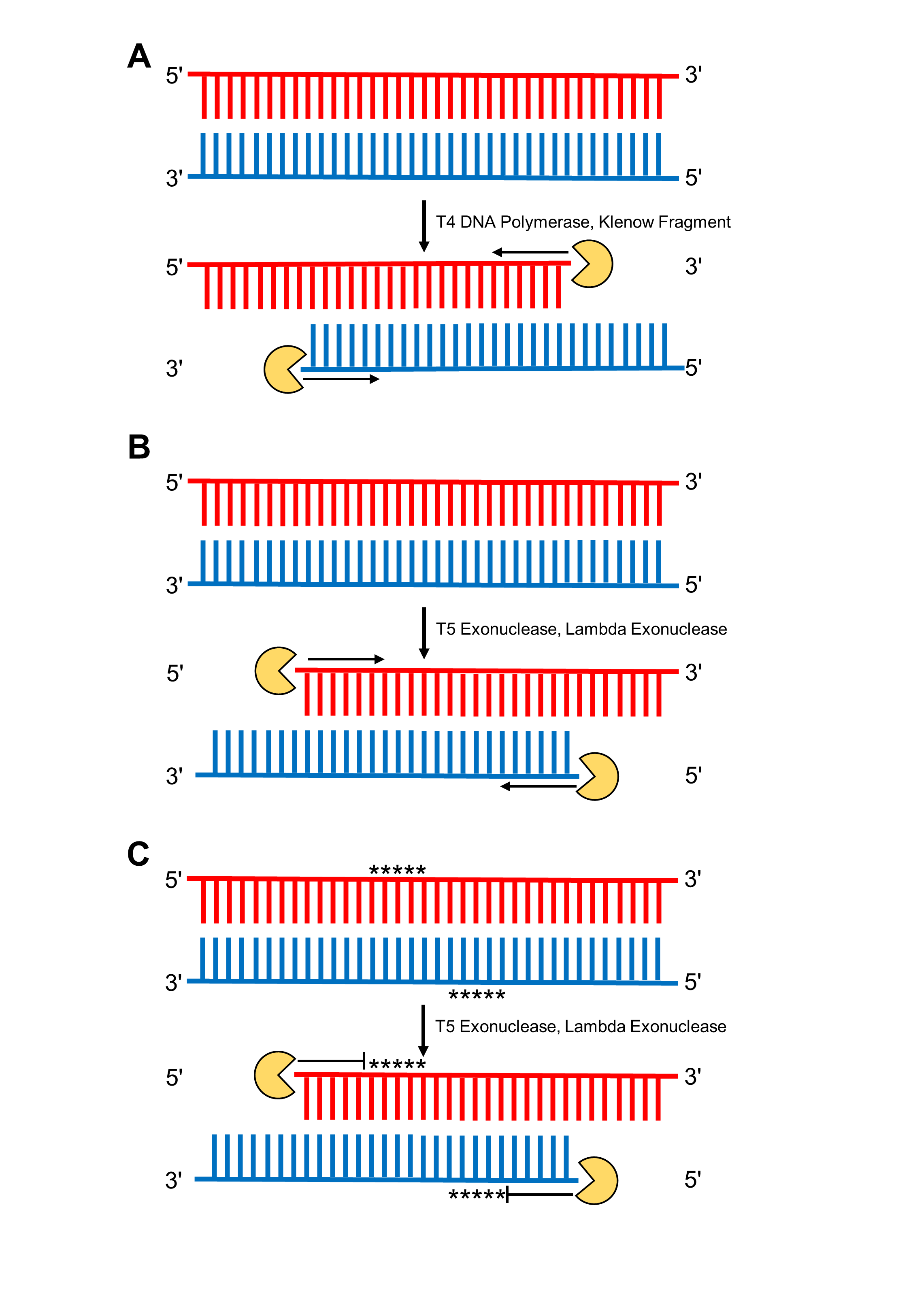

Supplement: S1 Fig — (A) T4 DNA polymerase and the large Klenow fragment, without supplementation of dNTPs, exhibit only 3’ →5’ exonuclease function, producing single-stranded DNA at the 5’ end. (B) T5 and lambda exonucleases have 5’ →3’ exonuclease activities, resulting in the production of 3’ overhangs. (C) Linear DNA with five tandem PTs defies the activities of exonuclease. Asterisks indicate PTs. (TIF) [file pone.0318015.s001.tif]

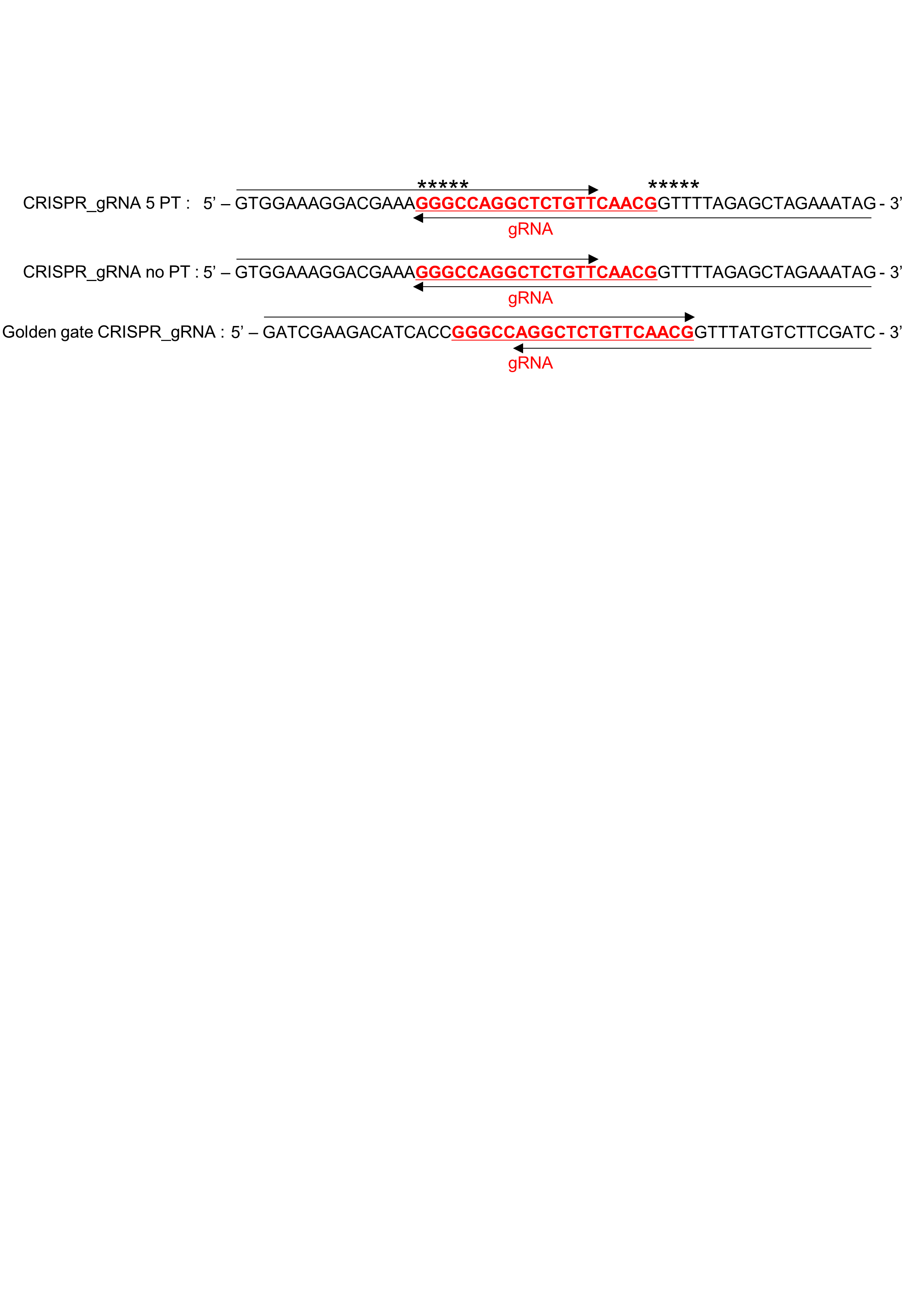

Supplement: S2 Fig — The primer dimers underwent PCR. Asterisks indicate the position and number of PT modifications, while red capitals represent gRNA sequences. (TIF) [file pone.0318015.s002.tif]

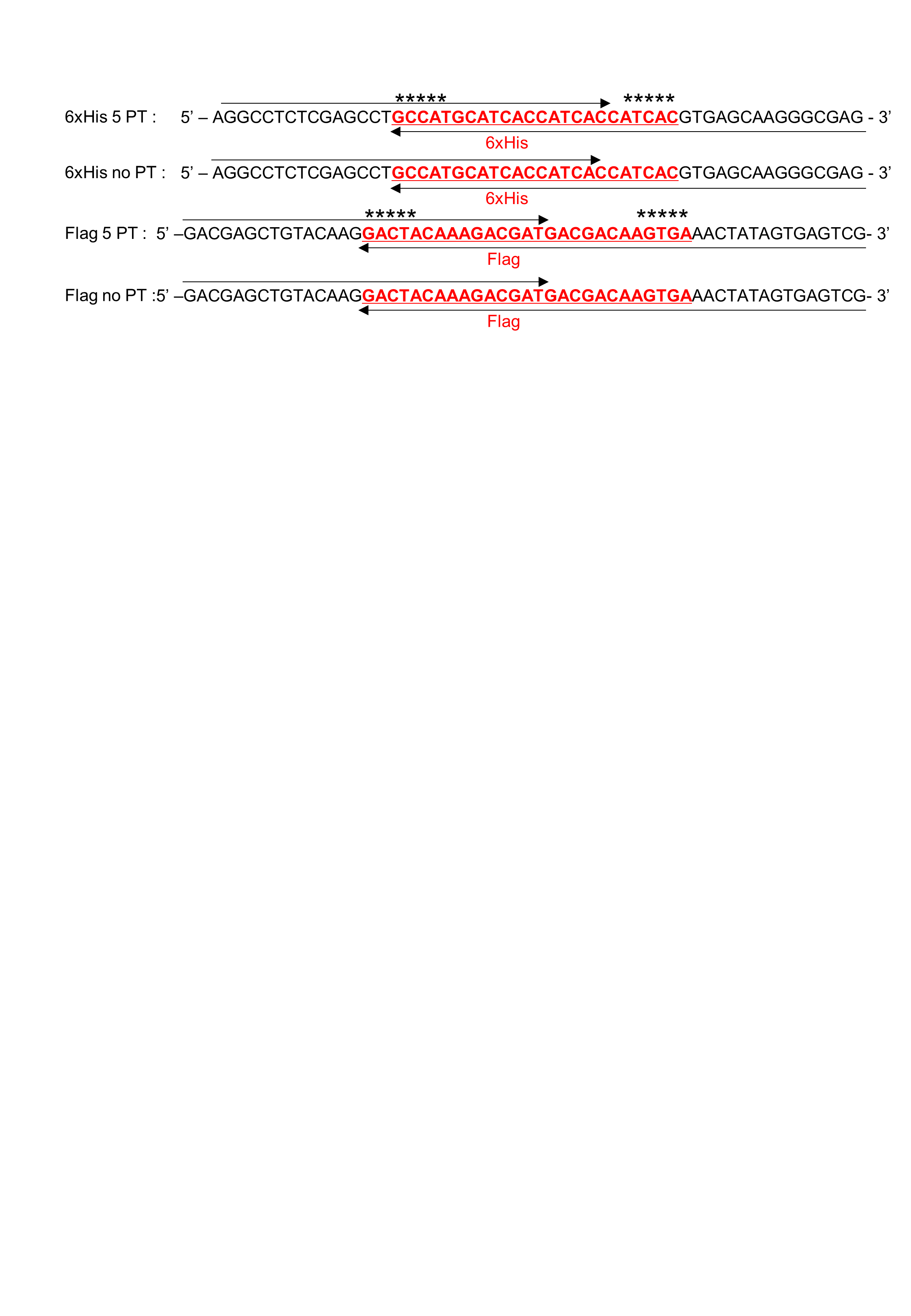

Supplement: S3 Fig — Asterisks represent PT-modified nucleotides, while red uppercase letters indicate sequences encoding 6xHis or Flag epitope. (TIF) [file pone.0318015.s003.tif]

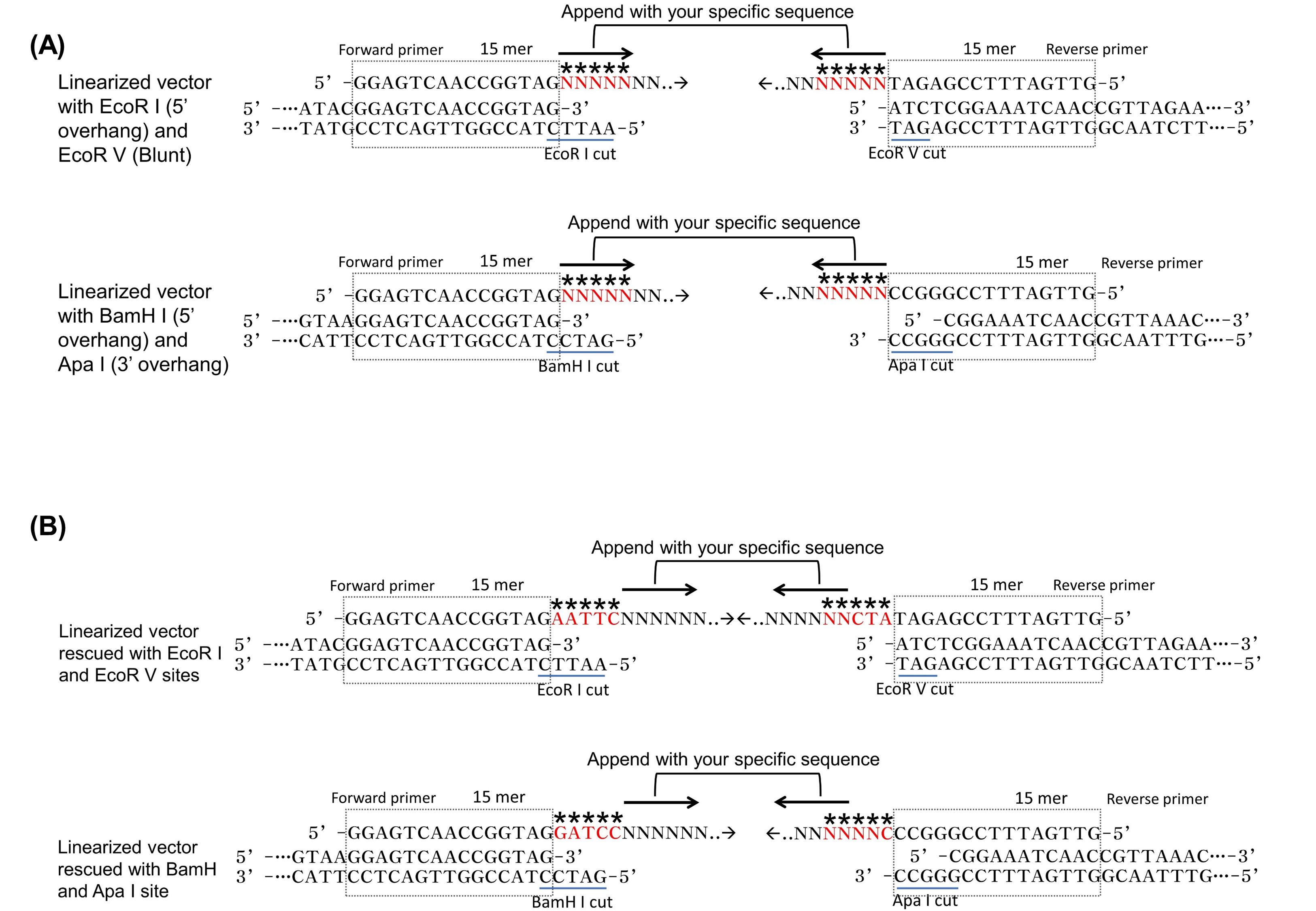

Supplement: S4 Fig — (A) The linearized vector, digested with the indicated REs (EcoRI, BamHI producing 5’ overhangs; ApaI producing 3’ overhangs; and EcoRV producing blunt ends), serves as the acceptor of the PCR products. Arrows represent primers, while dotted boxes denote the 15 bp complementary sequences between the termini of the vector and the PCR product. N stands for the sequences specific to the desired insert. Five consecutive asterisks above the blue ’N’ letters represent the positions of PT internucleotide linkages. After subcloning into the vector, the RE recognition sites may inevitably be erased. (B) The RE sites can be restored by adding appropriate nucleotides to the primers. The red capital letters represent the sequences for the indicated RE sites. (TIF) [file pone.0318015.s004.tif]
