# Supplementary material for: DAPE cloning with modified primers for producing designated lengths of 3’ single-stranded ends in PCR products
Source: PLoS One. 2025 Feb 13;20(2):e0318015. doi: 10.1371/journal.pone.0318015 (PMC11825038; doi:10.1371/journal.pone.0318015)
Supplement: S1 Table — (PDF) [file pone.0318015.s005.pdf]

S1 Table. Composition of the T5 storage solution and the 5x T5 reaction buffer. They should be stored at -20°C.

**T5 Exonuclease Storage solution**	**5x T5 reaction buffer**
50 mM Tris-HCl pH 7.5	0.5 M Tris-HCl pH 7.5
100 mM NaCl	50 mM $MgCl_2$
1 mM DTT	25% PEG 8000
0.1 mM EDTA	50 mM DTT
50% Glycerol	
0.1% Triton X-100	
