# Supplementary material for: DAPE cloning with modified primers for producing designated lengths of 3’ single-stranded ends in PCR products
Source: PLoS One. 2025 Feb 13;20(2):e0318015. doi: 10.1371/journal.pone.0318015 (PMC11825038; doi:10.1371/journal.pone.0318015)
Supplement: S2 Table — (PDF) [file pone.0318015.s006.pdf]

S2 Table. List of primers used for the experiments in Figures 1 and 2. Nucleotides labeled with an asterisk in square brackets indicate PT modification.

| | |
|---|---|
| 5'-Phophate 15mer 15mer 1 F | [Phosphate]CAGCCCGACCGCTGCGCCTTATCCGGTAAC |
| 5'-Phophate 15mer 15mer 2 R | [Phosphate]TTTTCGGGGAAATGTGCGCGGAACCCCTAT |
| 5'-Phophate 15mer 15mer 3 F | [Phosphate]ATAGGGGTTCCGCGCACATTTCCCCGAAAA |
| 5'-Phophate 15mer 15mer 4 R | [Phosphate]GTTACCGGATAAGGCGCAGCGGTCGGGCTG |
| 5'-Phophate 15mer 5PT 10mer 1 F | [Phosphate]CAGCCCGACCGCTGC[G*C*C*T*T*]ATCCGGTAAC |
| 5'-Phophate 15mer 5PT 10mer 2 R | [Phosphate]TTTTCGGGGAAATGT[G*C*G*C*G*]GAACCCCTAT |
| 5'-Phophate 15mer 5PT 10mer 3 F | [Phosphate]ATAGGGGTTCCGCGC[A*C*A*T*T*]TCCCCGAAAA |
| 5'-Phophate 15mer 5PT 10mer 4 R | [Phosphate]GTTACCGGATAAGGC[G*C*A*G*C*]GGTCGGGCTG |
| 15mer 5PT 10mer 1 F | CAGCCCGACCGCTGC[G*C*C*T*T*]ATCCGGTAAC |
| 15mer 5PT 10mer 2 R | TTTTCGGGGAAATGT[G*C*G*C*G*]GAACCCCTAT |
| 15mer 5PT 10mer 3 F | ATAGGGGTTCCGCGC[A*C*A*T*T*]TCCCCGAAAA |
| 15mer 5PT 10mer 4 R | GTTACCGGATAAGGC[G*C*A*G*C*]GGTCGGGCTG |
| 15mer 15mer 1 F | CAGCCCGACCGCTGCGCCTTATCCGGTAAC |
| 15mer 15mer 2 R | TTTTCGGGGAAATGTGCGCGGAACCCCTAT |
| 15mer 15mer 3 F | ATAGGGGTTCCGCGCACATTTCCCCGAAAA |
| 15mer 15mer 4 R | GTTACCGGATAAGGCGCAGCGGTCGGGCTG |
