# Supplementary material for: DAPE cloning with modified primers for producing designated lengths of 3’ single-stranded ends in PCR products
Source: PLoS One. 2025 Feb 13;20(2):e0318015. doi: 10.1371/journal.pone.0318015 (PMC11825038; doi:10.1371/journal.pone.0318015)
Supplement: S3 Table — (PDF) [file pone.0318015.s007.pdf]

S3 Table. List of primers used for the experiments in Figures 3 and 4. Nucleotides labeled with an asterisk in square brackets indicate PT modification.

5PT 6xHis F	AGGCCTCTCGAGCCT[C*A*T*C*A*]CCATCACCAT
5PT 6xHis R	CGACTCACTATAGTT[G*T*G*A*T*]GGTGATGGTGATG
3PT 6xHis F	AGGCCTCTCGAGCCT[C*A*T*]CACCATCACCAT
3PT 6xHis R	CGACTCACTATAGTT[G*T*G*]ATGGTGATGGTGATG
1PT 6xHis F	AGGCCTCTCGAGCCT[C*]ATCACCATCACCAT
1PT 6xHis R	CGACTCACTATAGTT[G*]TGATGGTGATGGTGATG
no PT 6xHis F	AGGCCTCTCGAGCCTCATCACCATCACCAT
no PT 6xHis R	CGACTCACTATAGTTGTGATGGTGATGGTGATG
