# Supplementary material for: DAPE cloning with modified primers for producing designated lengths of 3’ single-stranded ends in PCR products
Source: PLoS One. 2025 Feb 13;20(2):e0318015. doi: 10.1371/journal.pone.0318015 (PMC11825038; doi:10.1371/journal.pone.0318015)
Supplement: S4 Table — (PDF) [file pone.0318015.s008.pdf]

S4 Table. List of primers used for the experiments in Figure 5. Nucleotides labeled with an asterisk in square brackets indicate PT modification.

5PT gRNA F	GTGGAAAGGACGAAA[G*G*G*C*C*]AGGCTCTGTT
5PT gRNA R	CTATTTCTAGCTCTA[A*A*A*C*C*]GTTGAACAGAGCCTGGCCC
no PT gRNA F	GTGGAAAGGACGAAAGGGCCAGGCTCTGTT
no PT gRNA R	CTATTTCTAGCTCTAAAACCGTTGAACAGAGCCTGGCCC
Golden gate gRNA F	GATCGAAGACATCACCGGGCCAGGCTCTGTTCAACG
Golden gate gRNA R	GATCGAAGACATAAACCGTTGAACAGAGCCT
