# Supplementary material for: DAPE cloning with modified primers for producing designated lengths of 3’ single-stranded ends in PCR products
Source: PLoS One. 2025 Feb 13;20(2):e0318015. doi: 10.1371/journal.pone.0318015 (PMC11825038; doi:10.1371/journal.pone.0318015)
Supplement: S5 Table — (PDF) [file pone.0318015.s009.pdf]

S5 Table. List of primers used for the experiments in Figure 6. Nucleotides labeled with an asterisk in square brackets indicate PT modification.

50mer no PT F	AGGCCTCTCGAGCCTCACATGAAGCAGCACGACTT
50mer no PT R	CGACTCACTATAGTTAAGTCGTGCTGCTTC
50mer 5 PT F	AGGCCTCTCGAGCCT[C*A*C*A*T*]GAAGCAGCACGACTT
50mer 5 PT R	CGACTCACTATAGTT[A*A*G*T*C*]GTGCTGCTTC
80mer no PT F	AGGCCTCTCGAGCCTCACATGAAGCAGCACGACTTCTTCAAGTCCGCCATGCCCG
80mer no PT R	CGACTCACTATAGTTACGTAGCCTTCGGGCATGGCGGACT
80mer 5 PT F	AGGCCTCTCGAGCCT[C*A*C*A*T*]GAAGCAGCACGACTTCTTCAAGTCCGCCATGCCCG
80mer 5 PT R	CGACTCACTATAGTT[A*C*G*T*A*]GCCTTCGGGCATGGCGGACT
no PT F	AGGCCTCTCGAGCCTCACATGAAGCAGCAC
5 PT F	AGGCCTCTCGAGCCT[C*A*C*A*T*]GAAGCAGCAC
110 mer no PT R	CGACTCACTATAGTTTCGTCCTTGAAGAAG
110 mer 5 PT R	CGACTCACTATAGTT[T*C*G*T*C*]CTTGAAGAAG
140 mer no PT R	CGACTCACTATAGTTTTCACCTCGGCGCGG
140 mer 5 PT R	CGACTCACTATAGTT[T*T*C*A*C*]CTCGGCGCGG
170 mer no PT R	CGACTCACTATAGTTATGCGGTTCACCAGG
170 mer 5 PT R	CGACTCACTATAGTT[A*T*G*C*G*]GTTCACCAGG
