# Supplementary material for: DAPE cloning with modified primers for producing designated lengths of 3’ single-stranded ends in PCR products
Source: PLoS One. 2025 Feb 13;20(2):e0318015. doi: 10.1371/journal.pone.0318015 (PMC11825038; doi:10.1371/journal.pone.0318015)
Supplement: S6 Table — (PDF) [file pone.0318015.s010.pdf]

S6 Table. List of primers used for the experiments in Figure 7. Nucleotides labeled with an asterisk in square brackets indicate PT modification.

| | |
|---|---|
| secondary structure Leadzyme no PT F | AGGCCTCTCGAGCCTGCGACCGAGCCA GCGAAAGTTGGGAGTCGCGCAGCTCTGGCCCGT |
| secondary structure Leadzyme no PT R | TCACTATAGTTCTAGGCGACTCCCAACTTTCGCTGGCTCG GTCGCTTAGAAAAACTCATC |
| secondary structure Leadzyme 5 PT F | AGGCCTCTCGAGCCT[G*C*G*A*C*]CGAGCCA GCGAAAGTTGGGAGTCGCGCAGCTCTGGCCCGT |
| secondary structure Leadzyme 5 PT R | TCACTATAGTTCTAG[G*C*G*A*C*]TCCCAACTTTCGCTGGC TCGGTCGCTTAGAAAAACTCATC |
