# Supplementary material for: DAPE cloning with modified primers for producing designated lengths of 3’ single-stranded ends in PCR products
Source: PLoS One. 2025 Feb 13;20(2):e0318015. doi: 10.1371/journal.pone.0318015 (PMC11825038; doi:10.1371/journal.pone.0318015)
Supplement: S7 Table — (PDF) [file pone.0318015.s011.pdf]

S7 Table. List of primers used for the experiments in Figure 8. Nucleotides labeled with an asterisk in square brackets indicate PT modification.

3piece 6xHis Start codon no PT F	AGGCCTCTCGAGCCTGCCATGCATCACCATCACCAT
3piece 6xHis Start codon 5 PT F	AGGCCTCTCGAGCCT[G*C*C*A*T*]GCATCACCATCACCAT
3piece 6xHis no PT R	CTCGCCCTTGCTCACGTGATGGTGATGGTGATG
3piece 6xHis 5 PT R	CTCGCCCTTGCTCAC[G*T*G*A*T*]GGTGATGGTGATG
3piece EGFP no PT F	CACCATCACCATCACGTGAGCAAGGGCGAG
3piece EGFP no PT R	ATCGTCTTTGTAGTCCTTGTACAGCTCGTC
3piece Flag no PT F	GACGAGCTGTACAAGGACTACAAAGACGAT
3piece Flag 5 PT F	GACGAGCTGTACAAG[G*A*C*T*A*]CAAAGACGAT
3piece Flag Stop codon no PT R	CGACTCACTATAGTTTCACTTGTCGTCATCGTCTTTGTAGTC
3piece Flag Stop codon 5 PT R	CGACTCACTATAGTT[T*C*A*C*T*]TGTCGTCATCGTCTTTGTAGTC
