# Supplementary material for: DAPE cloning with modified primers for producing designated lengths of 3’ single-stranded ends in PCR products
Source: PLoS One. 2025 Feb 13;20(2):e0318015. doi: 10.1371/journal.pone.0318015 (PMC11825038; doi:10.1371/journal.pone.0318015)
Supplement: S1 Raw images — (PDF) [file pone.0318015.s012.pdf]

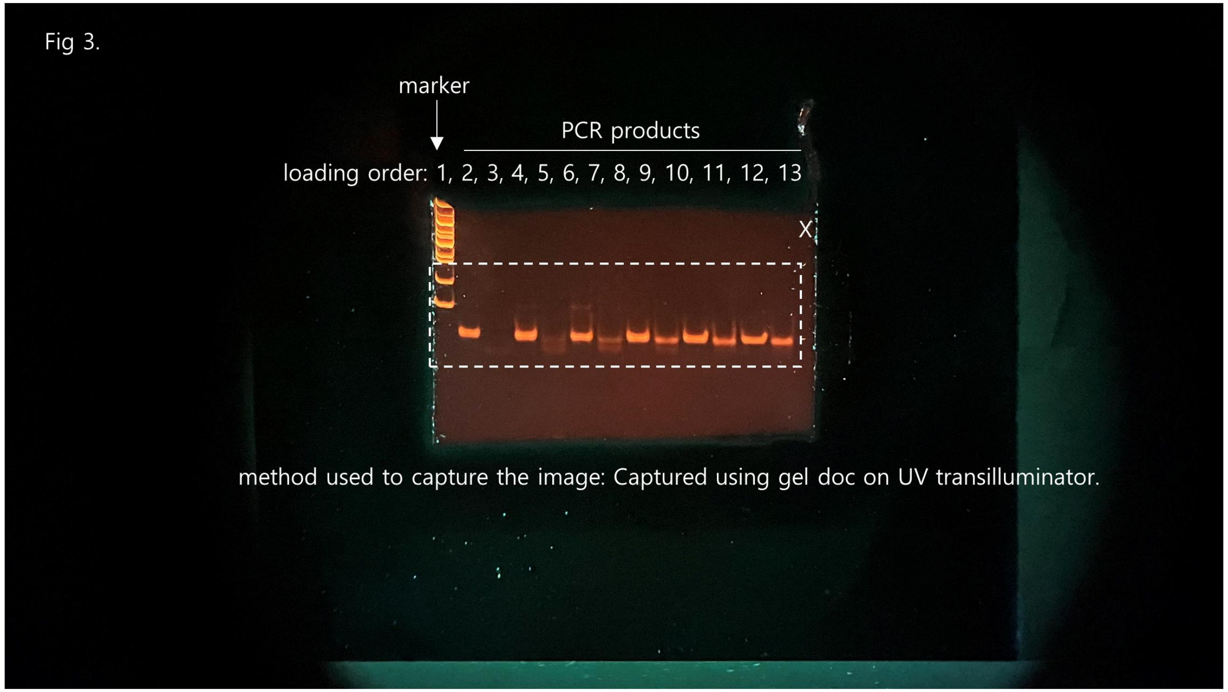
Fig 3.
marker
PCR products
loading order: 1, 2, 3, 4, 5, 6, 7, 8, 9, 10, 11, 12, 13
X
method used to capture the image: Captured using gel doc on UV transilluminator.

Fig 7.

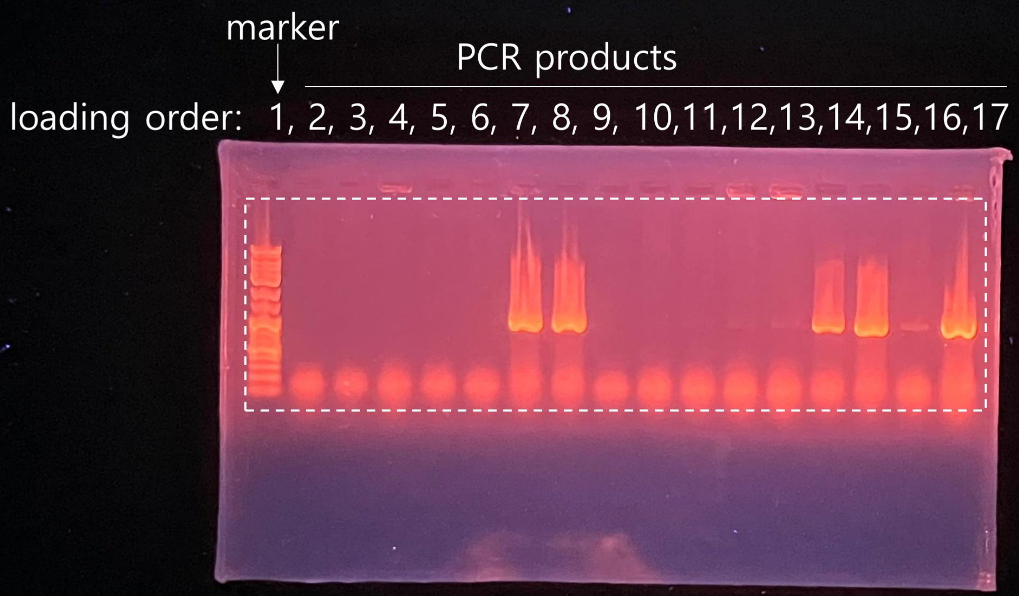

method used to capture the image: Captured using gel doc on UV transilluminator.

Fig 7.

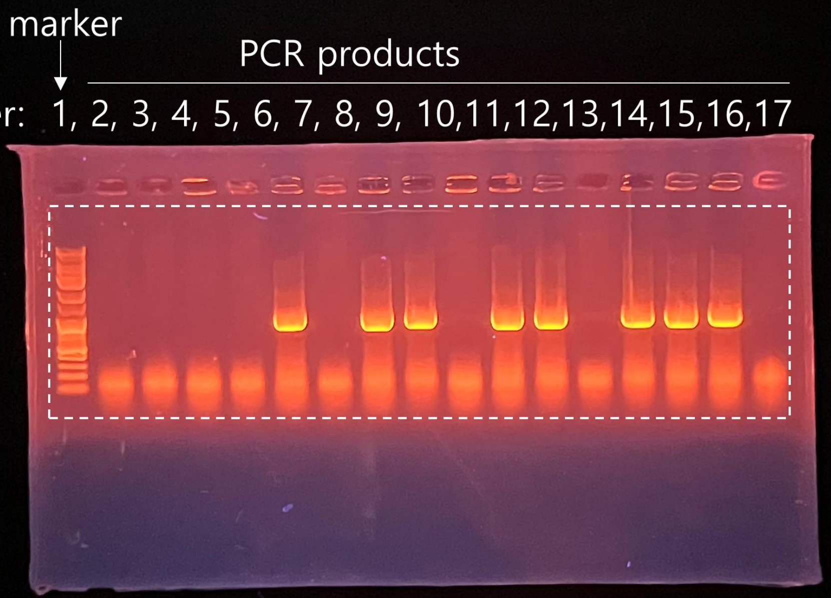

method used to capture the image: Captured using gel doc on UV transilluminator.

Fig 8.

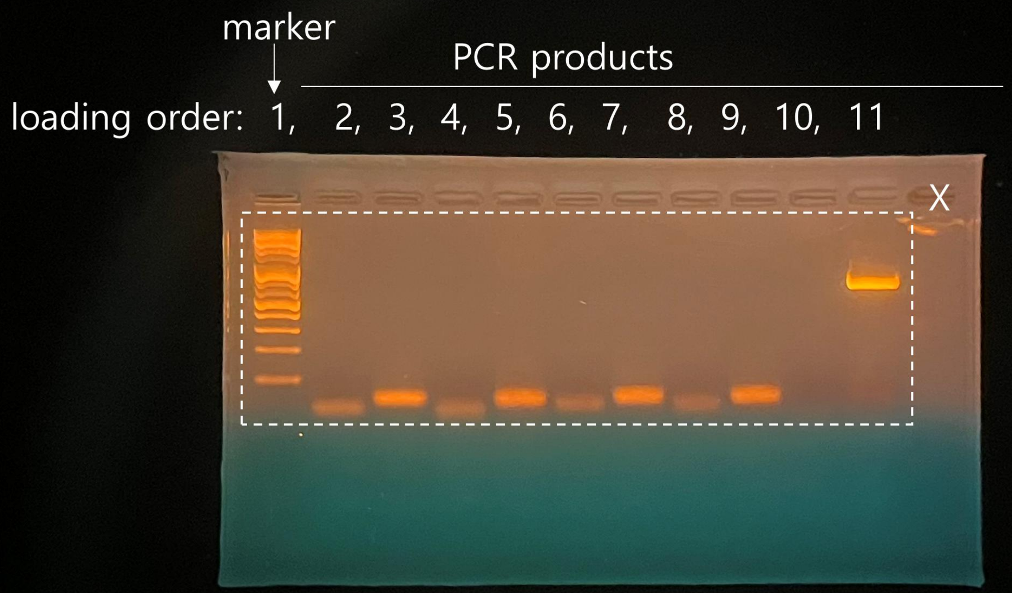

method used to capture the image: Captured using gel doc on UV transilluminator.
